# Supplementary material for: A Novel Role for Plasminogen Activator Inhibitor Type-2 as a Hypochlorite-Resistant Serine Protease Inhibitor and Holdase Chaperone
Source: Cells. 2022 Mar 29;11(7):1152. doi: 10.3390/cells11071152 (PMC8997907; doi:10.3390/cells11071152)
Supplement: Supplementary file 1 [file cells-11-01152-s001.zip › cells-1644208-supplementary.pdf]

## Supplementary Material


| | Page |
|---|---|
| Figure S1 | 2 |
| Figure S2 | 3 |
| Figure S3 | 4 |
| Figure S4 | 5 |
| Figure S5 | 6 |
| Table S1 | 7 |
| Table S2 | 8 |
| Table S3 | 8 |

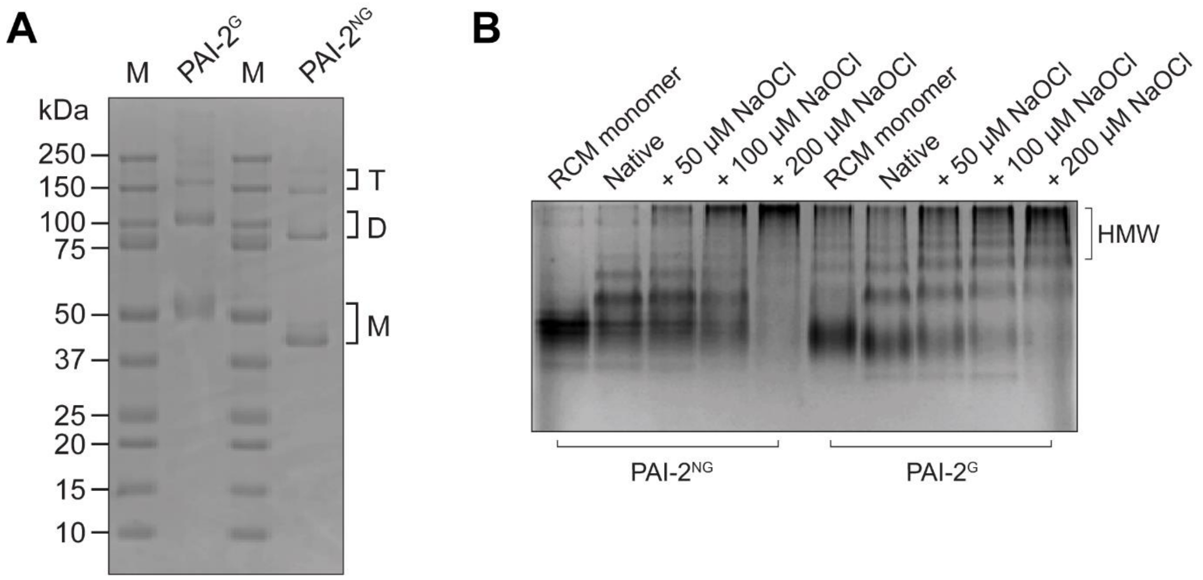


**Figure S1. SDS-PAGE and native PAGE analyses of PAI-2$^{G}$ and PAI-2$^{NG}$.** (A) PAI-2$^{G}$ and PAI-2$^{NG}$ were separated by non-reducing SDS-PAGE using a 4–12% Bis-Tris gel. Protein molecular weight standards (lane M) are included for comparison, with sizes as indicated in kDa. Positions of PAI-2$^{G}$ and PAI-2$^{NG}$ monomers (M), dimers (D) and trimers (T) are marked. (B) PAI-2$^{G}$ or PAI-2$^{NG}$ pre-treated with 0–200 μM NaOCl were separated by native PAGE using an 8% Tris-glycine gel. A reduced, S-carboxymethylated sample (RCM monomer) is included as a standard to define the migratory position of the monomer for each PAI-2$^{G}$ and PAI-2$^{NG}$. High molecular weight oligomers and complexes (HMW) are marked. Note the lower isoelectric point of PAI-2$^{G}$ (pI=4.4), versus PAI-2$^{NG}$ (pI=5), which affects migration under native PAGE conditions (approximate running pH=8).

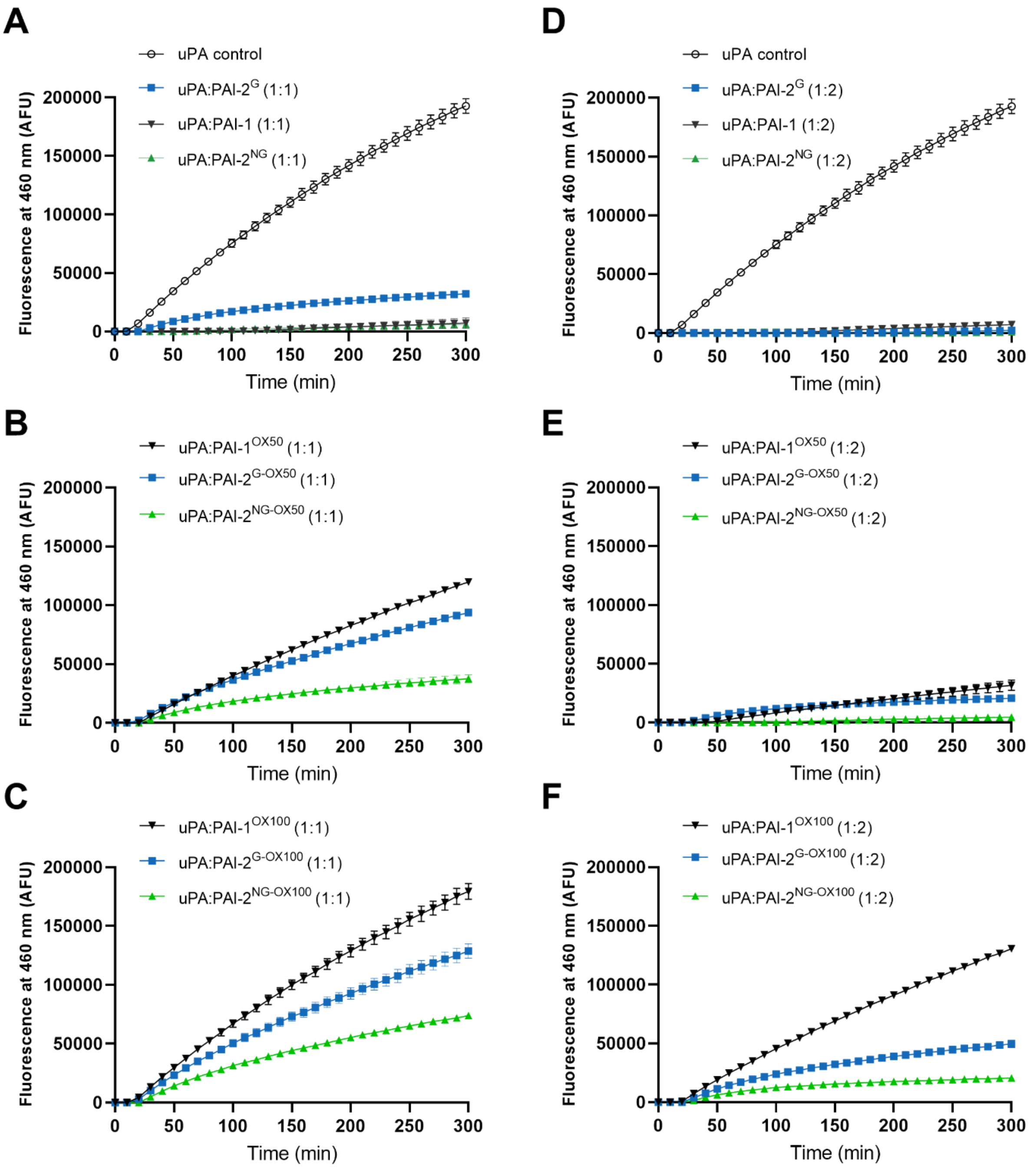


**Figure S2. Effect of hypochlorite treatment on uPA inhibitory activity of PAIs at different molar ratios of PAI to uPA.** Representative kinetic uPA activity plots showing the inhibitory effect of (A) Native PAIs, (B) 50 μM NaOCl pre-treated (OX50) PAIs and (C) 100 μM NaOCl pre-treated (OX100) PAIs at a 1:1 molar ratio of uPA:PAI; or (D) Native PAIs, (E) OX50 PAIs and (F) 100OX PAIs at a 1:2 molar ratio of uPA:PAI. Data points show the mean fluorescence ± SD for triplicate determinations at each time point. The fluorescence of uPA control (no PAIs) was included as a control. The average rate of change in fluorescence is presented in Figure 2 of the main article.

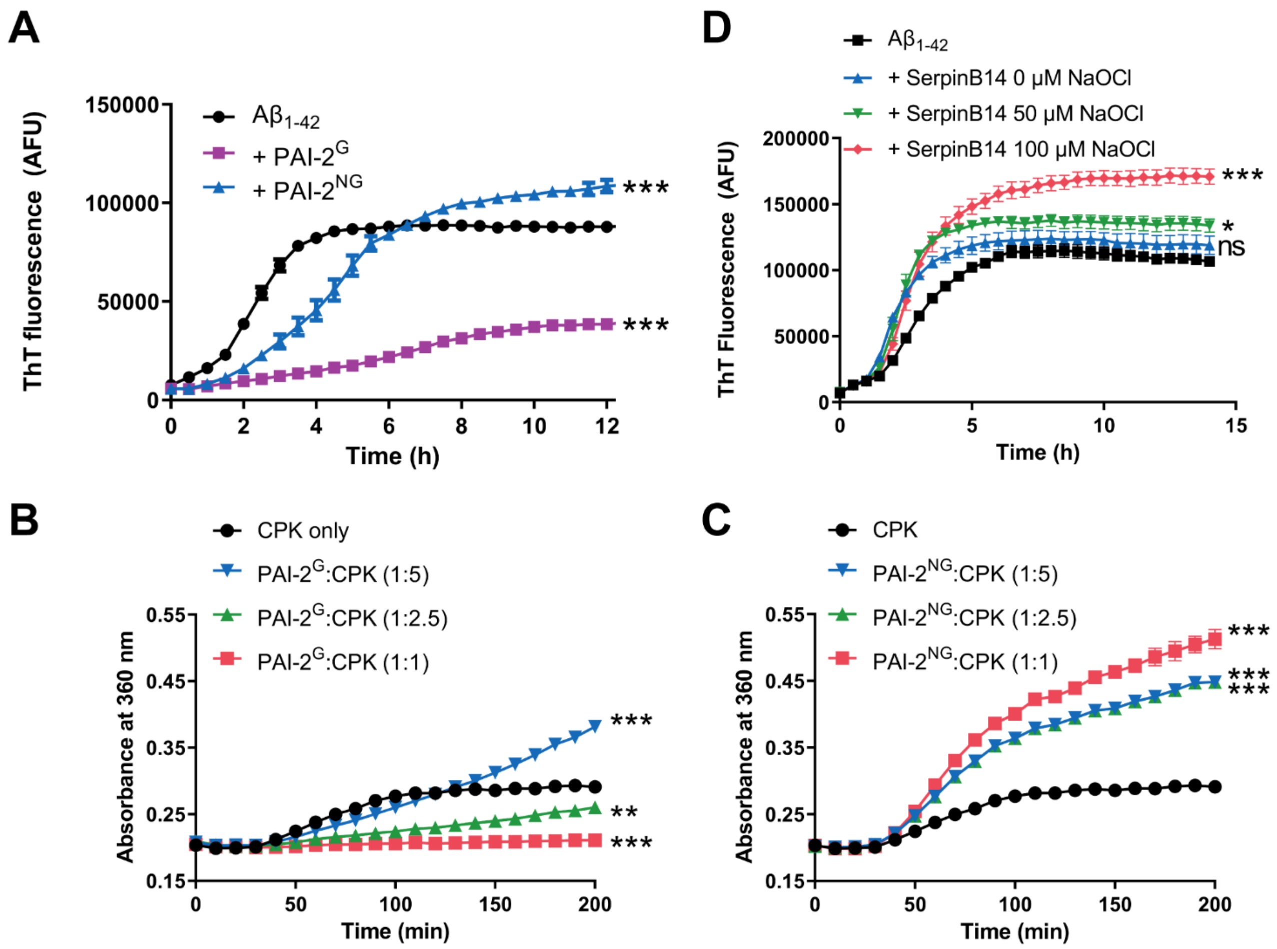


**Figure S3.** Effect of PAI-2$^{G}$ and PAI-2$^{NG}$ on the aggregation of A$\beta_{1\text{–}42}$ and creatine phosphokinase, and the effect of SerpinB14 on the aggregation of A$\beta_{1\text{–}42}$ following hypochlorite treatment. (A) A$\beta_{1\text{–}42}$ (3 μM) was incubated with 25 μM ThT in PBS at 28°C ± 20 μg/mL of PAI-2$^{G}$ or PAI-2$^{NG}$. (B and C) Creatine phosphokinase (CPK; 6.5 μM) was incubated at 43°C with shaking ± (B) PAI-2$^{G}$ or (C) PAI-2$^{NG}$ at molar ratios of 1:1. 1:2.5 or 1:5 (PAI-2:CPK). Turbidity indicative of CPK aggregation was monitored by measuring absorbance at 360 nm of the solution. (D) A$\beta_{1\text{–}42}$ was incubated as before except ± SerpinB14 that had been pre-treated with 0–100 μM hypochlorite at a 1:10 molar ratio (SerpinB14:A$\beta_{1\text{–}42}$). All data are means ± SEM (n=3) and are representative of three independent experiments. One-way ANOVA with post-hoc Tukey's HSD was conducted on the assay endpoints to identify non-significant (ns) and significant differences (*** = $P < 0.001$; ** = $P < 0.01$; * = $P < 0.05$). Statistical results are shown based on comparisons with A$\beta_{1\text{–}42}$ or CPK only.

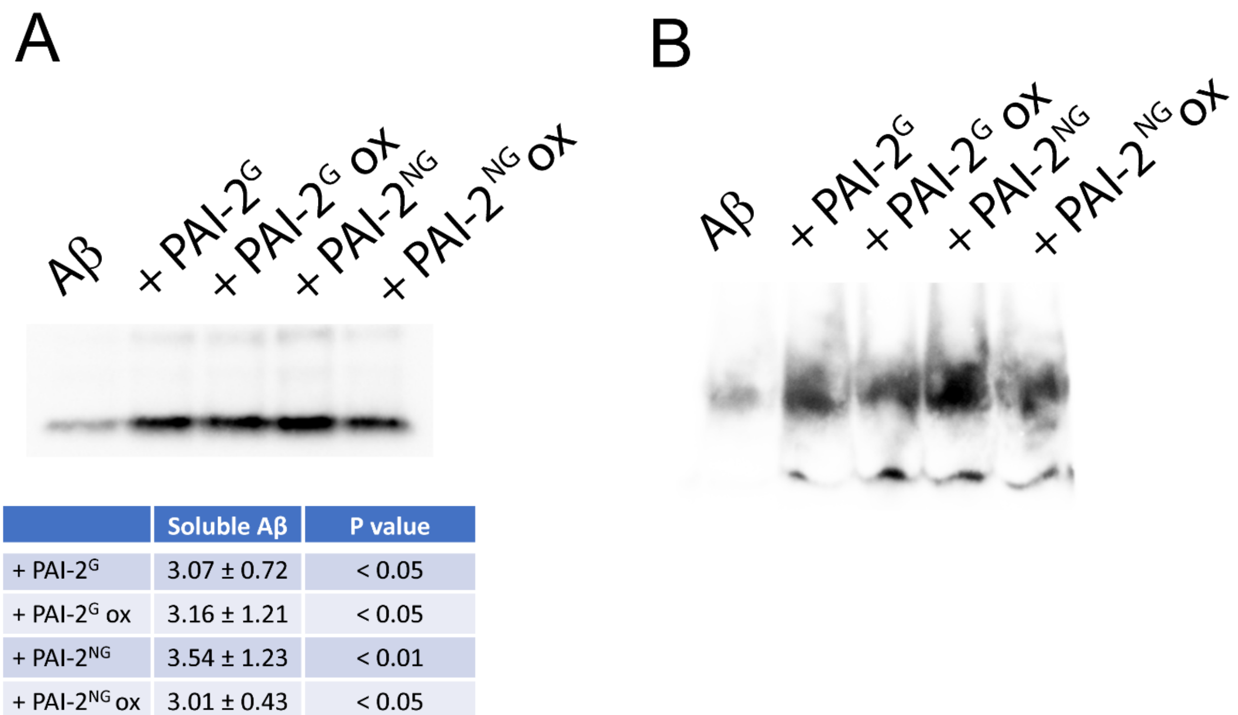


| | Soluble Aβ | P value |
|---|---|---|
| + PAI-2$^{G}$ | 3.07 ± 0.72 | < 0.05 |
| + PAI-2$^{G}$ ox | 3.16 ± 1.21 | < 0.05 |
| + PAI-2$^{NG}$ | 3.54 ± 1.23 | < 0.01 |
| + PAI-2$^{NG}$ ox | 3.01 ± 0.43 | < 0.05 |

**Figure S4**. Effect of PAI-2 on the solubility of Aβ1-42 as assessed by (A) native Western Blot analysis and (B) streptavadin-biotin pull down assay. PAI-2$^{G}$ and PAI-2$^{NG}$ were pre-treated with 200 μM NaOCl in PBS and then extensively dialysed to generate PAI-2$^{G}$ox and PAI-2$^{NG}$ox, respectively (see Methods 2.3). All samples of PAI-2 were then biotinylated following the manufacturer's instructions (Sigma-Aldrich). A$\beta_{1\text{-}42}$ (5 μM) was incubated with 25 μM ThT in PBS at 30°C ± 1 μM PAI-2$^{G}$, PAI-2$^{NG}$, PAI-2$^{G}$ox or PAI-2$^{NG}$ox overnight. Following centrifugation, the soluble protein fraction was recovered and subjected to native Western blot analysis for A$\beta_{1\text{-}42}$ detected using a monoclonal anti-Aβ1-42 antibody (W02). Panel (A) shows a representative Western blot image of A$\beta_{1\text{-}42}$. Densitometry analysis was performed and the mean soluble Aβ1-42 relative to the control sample containing Aβ1-42 alone is reported (n = 3 ± SD). All forms of PAI-2 increased the solubility of Aβ1-42, compared to Aβ1-42 alone, as determined using one-way ANOVA and post-hoc Tukey's HSD analysis. However, there was no difference between the different PAI-2 forms under the conditions used. Panel (B) shows a Western Blot of A$\beta_{1\text{-}42}$ pulled down with biotinylated PAI-2 recovered using Dynabeads™ MyOne™ Streptavidin C1 according to the manufacturer's instructions (Invitrogen). Washing was performed using PBS, pH 7.4 containing 0.01% [w/v] BSA to minimise the non-specific binding of Aβ1-42 to the beads. The recovered protein was separated by reducing SDS-PAGE using a 4–12% Bis-Tris gel prior to Western blot analysis.

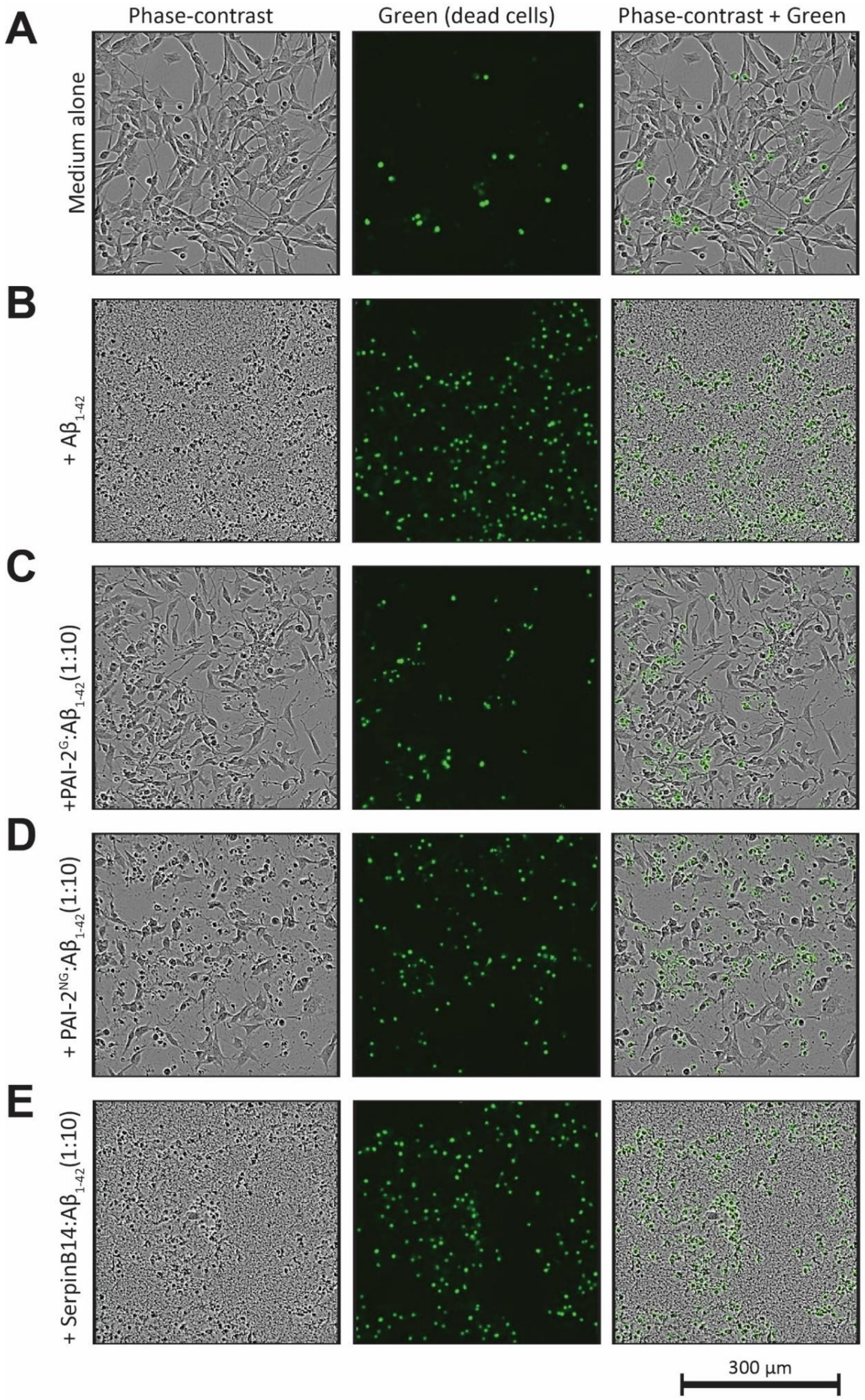


**Figure S5**. Representative photomicrographs showing the effect of PAI-2 on $A\beta_{1–42}$-induced cytotoxicity and morphology of SH-SY5Y cells. Images taken at 48 h timepoint from a cytotoxicity experiment as described in Figure 3F for (*A*) medium alone control, (*B*) $A\beta_{1-42}$,(*C*) PAI-$2^{G}$:$A\beta_{1-42}$ (1:10 molar ratio), (*D*) PAI-$2^{NG}$:$A\beta_{1-42}$ (1:10 molar ratio) and (*E*) SERPINB14:$A\beta_{1-42}$ (1:10 molar ratio).

**Table S1.** Summary of demographic and clinical characteristics of the subjects whose placentas were analysed as part of this study, along with immunohistochemical scores.

| Variable | Severe Preeclampsia n=15 | Idiopathic Preterm Birth n=15 | p value |
|---|---|---|---|
| Maternal age, years * | 25 ± 9 | 29 ± 8 | 0.146 |
| Race/ethnicity † | | | |
| Non-Hispanic White | 7 (47) | 8 (53) | |
| Non-Hispanic Black | 6 (40) | 6 (40) | 0.381 |
| Hispanic | 2 (13) | 0 (0) | |
| Other | 0 (0) | 1 (7) | |
| Nulliparity ‡ | 11 (73) | 7 (47) | 0.264 |
| Systolic blood pressure * | 169 ± 20 | 118 ± 13 | <0.001 |
| Diastolic blood pressure * | 99 ± 10 | 67 ± 6 | <0.001 |
| HELLP manifestations ‡ | 4 (27) | 0 (0) | 0.100 |
| Fetal growth restriction ‡ | 6 (40) | 0 (0) | 0.017 |
| Neurological symptoms including headache ‡ | 7 (47) | 0 (0) | 0.006 |
| Seizures ‡ | 1 (7) | 0 (0) | 1.000 |
| Gestational age at delivery, weeks * | 30 ± 2 | 30 ± 2 | 0.823 |
| Medically indicated delivery for preeclampsia ‡ | 15 (100) | 0 (0) | <0.001 |
| PPROM ‡ | 6 (40) | 0 (0) | 0.017 |
| Cesarean delivery ‡ | 13 (87) | 8 (53) | |
| Birthweight, grams * | 1,269 ± 458 | 1,597 ± 408 | 0.048 |
| Newborn sex ‡ | | | |
| Female | 8 (53) | 10 (67) | 0.710 |
| Male | 7 (47) | 5 (33) | |
| 1 min Apgar § | 6 [5 – 7] | 7 [5 – 8] | 0.344 |
| 5 min Apgar § | 8 [8 – 9] | 9 [8 – 9] | 0.162 |
| PAI-2 staining score § | 1 [0-4] | 2 [1-5] | 0.246 |
| PAI-2-staining score distribution † | | | |
| Absent or weak (0-1) | 8 (53) | 4 (27) | 0.189 |
| Intermediate (2-3) | 2 (13) | 6 (40) | |
| Strong (4-5) | 5 (33) | 5 (33) | |

* Data presented as mean ± standard deviation and compared using Student t-test

† Data presented as n (%) and analysed by Chi-square test

‡ Data presented as n (%) and analysed by Fisher's exact test

§ Data presented as median [interquartile range] and analysed by Mann-Whitney test

Table S2: Summary of ThT fluorescence curves generated by incubating $A\beta_{1-42}$ ± PAI-2 as shown in Fig. 3A and B.

| | **Initial lag phase duration (h)** | **Maximum rate of aggregation (ΔThT AFU/h) n= 3 ± SD** | **Maximum THT fluorescence (AFU) n= 3 ± SD** |
|---|---|---|---|
| $A\beta_{1-42}$ | Negligible | 36,037.33 ± 4,353.51 | 63,225.80 ± 2,005.26 |
| PAI-$2^{G}$: $A\beta_{1-42}$ (1:10) | 2 | **7,483.70 ± 940.81 *** | 63,225.84 ± 5,034.73 * |
| PAI-$2^{G}$: $A\beta_{1-42}$ (1:7.5) | 2.4 | **3,982.13 ± 1,432.33** | 44,299.17 ± 16,177.31 ** |
| PAI-$2^{G}$: $A\beta_{1-42}$ (1:5) | 5 | **2,819.45 ± 436.27 **** | **27,566.84 ± 4,240.52 *** |
| PAI-$2^{G}$: $A\beta_{1-42}$ (1:2.5) | 5.5 | **124.11 ± 67.66 **** | **9,188.50 ± 2,158.28 *** |
| PAI-$2^{NG}$: $A\beta_{1-42}$ (1:10) | 0.5 | 23,868.11 ± 7,139.39 | 131,560.20 ± 27,517.30 |
| PAI-$2^{NG}$: $A\beta_{1-42}$ (1:7.5) | 1.5 | **17,336.33 ± 8,006.07** | 117,279.52 ± 43,669.01 |
| PAI-$2^{NG}$: $A\beta_{1-42}$ (1:5) | 2.5 | **5,074.67 ± 701.37** | 45,759.50 ± 5,459.84 |
| PAI-$2^{NG}$: $A\beta_{1-42}$ (1:2.5) | 4 | **2,674.57 ± 509.52** | **20,800.50 ± 1,919.77** |

Bold font denotes reduced compared to $A\beta_{1-42}$ alone; Tukey HSD $p < 0.05$. * or ** Denotes reduced compared to the corresponding sample containing PAI-$2^{NG}$ at the same molar ratio $p < 0.05$ and $p < 0.01$, respectively; Student's t-test

Table S3: Summary of ThT fluorescence curves generated by incubating $A\beta_{1-42}$ ± PAI-2 pre-treated with 0-200 μM NaOCl as shown in Fig. 3C and D.

| | **Initial lag phase duration (h)** | **Maximum rate of aggregation (ΔThT AFU/h) n= 3 ± SD** | **Maximum THT fluorescence (AFU) n= 3 ± SD** |
|---|---|---|---|
| $A\beta_{1-42}$ | Negligible | 20,207.33 ± 993.57 | 87,102.66 ± 1,245.23 |
| + PAI-$2^{G}$ 0 μM NaOCl | 2 | **4,879.87 ± 1,307.28** | **39,113.67 ± 1,079.35** |
| + PAI-$2^{G}$ 50 μM NaOCl | 2 | **2,961.39 ± 616.85** | **30,615.11 ± 5,361.45** |
| + PAI-$2^{G}$ 100 μM NaOCl | 2 | **2,763.52 ± 769.47** | **33,275.34 ± 2,473.78** |
| + PAI-$2^{G}$ 200 μM NaOCl | 2 | **4,079.08 ± 220.41** | **40,555.00 ± 2,351.17** |
| + PAI-$2^{NG}$ 0 μM NaOCl | 1.5 | **16,107.78 ± 377.20** | 109,826.37 ± 6,464.30 |
| + PAI-$2^{NG}$ 50 μM NaOCl | 2 | **4,347.00 ± 966.94 *** | **34,817.33 ± 6,261.53 *** |
| + PAI-$2^{NG}$ 100 μM NaOCl | 2 | **4,888.67 ± 279.21 *** | **41,483.67 ± 1,749.58 *** |
| + PAI-$2^{NG}$ 200 μM NaOCl | 2 | **3,457.89 ± 329.73 *** | **31,049.67 ± 3,549.68 *** |

Bold font denotes reduced compared to $A\beta_{1-42}$ alone; Tukey HSD $p < 0.05$. * Denotes reduced compared to the corresponding sample containing PAI-$2^{NG}$ 0 μM NaOCl; $p < 0.01$, Student's t-test
